# Evaluation of the effect of essential oils of Cinnamomum zeylanicum and Eucalyptus globulus against Salmonella Enteritidis and Salmonella Gallinarum in broilers

**DOI:** 10.5455/javar.2025.l970

**Published:** 2025-12-25

**Authors:** Sidra Yasmin, Muhammad Taimoor, Hira Noor, Muhammad Nawaz, Aftab Ahmad Anjum, Kamran Ashraf

**Affiliations:** 1 Institute of Microbiology, University of Veterinary and Animal Sciences, Lahore, Pakistan; 2 Research and Development Division, Veterinary Research Institute, Lahore, 54810, Pakistan; 3 Department of Parasitology, University of Veterinary and Animal Sciences, Lahore, 54000, Pakistan

**Keywords:** Broilers, Cinnamomum zeylanicum, Eucalyptus globulus, Essential oils, Salmonella Enteritidis, Salmonella Gallinarum

## Abstract

**Objective:**

This study intended to assess the effect of *Cinnamomum zeylanicum* and *Eucalyptus globulus* essential oils (Eos) as an alternative to antibiotics on *Salmonella* spp. infection, antibiotic growth promotion, and immunomodulation in experimentally infected broiler chickens.

**Materials and Methods:**

Broiler chicks (*n* = 135) were randomly and equally divided into nine groups. From day 1, experimental groups were dietary supplemented with *C*. *zeylanicum* or *E*. *globulus* EOs. *Salmonella* Enteritidis (SE) and *Salmonella* Gallinarum (SG) counts in droppings, growth performance (weight gain, feed conversion ratio), humoral immune response to Newcastle Disease Virus (NDV) and Infectious Bursal Disease Virus (IBDV), and gut morphology were measured in birds.

**Results:**

The antibiotic groups and the positive control group recorded significantly higher SE and SG counts compared to the EO groups. *Cinnamomum zeylanicum* recorded the highest reduction in SE and SG counts. Birds fed EOs gained weight much faster on day 35 and improved their NDV and IBDV titers. Supplementation with the EO resulted in the lengthening of villi and an increase in mucosal surface area in various intestinal sections, including the duodenum, jejunum, and ileum, as observed under the microscope, indicating an improvement in gut function.

**Conclusion:**

*Cinnamomum zeylanicum* and *E*. *globulus* EOs both exhibited a high *in vivo* anti-*Salmonella* effect, better growth performance, and improved immune and gut conditions in broilers. These results support the use of plant-based EOs as natural and antibiotic-free alternatives for controlling *Salmonella* infections in chicken.

## Introduction

Poultry meat is among the most widely consumed sources of animal protein and has remained a significant contributor to food security, particularly in developing nations [1]. In Pakistan, the poultry industry accounts for approximately 40% of total meat production, sustaining over 1.5 million people [2]. Although this has improved, several health issues persist in the industry, and one of the most severe is salmonellosis [3]. In addition to increasing morbidity and mortality rates in poultry [4], *Salmonella* is a zoonotic bacterium that poses a threat to public health by transmitting through contaminated poultry products [5].

Antibiotics have been widely used in poultry farms to treat and prevent bacterial infections, as well as to promote growth [6,7]. Nevertheless, they have contributed to the current worldwide issue of antimicrobial resistance [8] and to the occurrence of drug residues in meat, which causes food safety concerns [9]; that is why the need to research safer and more sustainable alternatives to antibiotics is pressing indeed. Some of the methods that have been investigated include nanoparticles, probiotics, prebiotics, pharmaceutical plants, and phytogenic feed additives [10,11]. Among them, the increasing popularity of essential oils (EOs) has been attributed to their natural antimicrobial, antioxidant, and growth-promoting properties.

EOs are volatile plant compounds that are often based on terpenes, aldehydes, phenolic compounds, and flavonoids [12]. They are able to disrupt the cell walls of bacteria, leading to leakage of cellular content, interference with enzymes, and even disruption of quorum sensing and biofilm formation [13]. EOs have been demonstrated to cause a decline in gut pathogens and enhance nutrient intake, as well as enhance the immune system in poultry [14–16]. Their anti-inflammatory and antioxidant properties also reinforce their capabilities as promising alternatives to antibiotic growth promoters [17,18].

Among many other EOs, cinnamon (*Cinnamomum* *zeylanicum*) and eucalyptus (*Eucalyptus globulus*) stand out. Cinnamon oil contains cinnamaldehyde, which is a potent antimicrobial and antifungal agent. It has been attributed to improved gut health and improved growth in poultry [19]. While eucalyptus oil contains 1,8-cineole (eucalyptol), phenolic acids, and flavonoids. These components make it anti-bacterial, anti-oxidant, and anti-inflammatory [20].

There is limited information available on the use of cinnamon and eucalyptus oils against *Salmonella* Enteritidis (SE) and *Salmonella* Gallinarum (SG) in broilers under local production conditions, despite some encouraging reports. With this consideration, the current experiment aimed to assess the impact of *C*. *zeylanicum* and *E*. *globulus* EOs on growth performance, intestinal morphology, *Salmonella* shedding, and humoral immune response to Newcastle Disease (ND) and Infectious Bursal Disease (IBD) vaccines in experimentally challenged broilers.

## Materials and Methods

### Ethics approval

The research was conducted in accordance with the ethical standards established by the Ethical Review Committee of the University of Veterinary and Animal Sciences (UVAS), Lahore, Pakistan [Approval No. DR/1103, dated October 11, 2017].

### Study design

The EOs of *C. zeylanicum* and *E. globulus* used in this study were from the same batch that was previously extracted, characterized, and evaluated for antimicrobial activity against SEand SGin our earlier published study [21]. Summarily, the plant materials were purchased at local herbal markets in Lahore, Pakistan, and verified at the Department of Botany at Government College University, Lahore. The plant parts were dried in the shade and reduced to a fine powder. The EOs were collected through the steam distillation process using a Clevenger-type distillation apparatus. Here, 300 gm of the dried vegetative material were distilled using 600 ml of distilled water within 4 to 5 h. The oils were extracted and dehydrated with anhydrous sodium sulfate, and afterwards placed in amber glass bottles at 4°C awaiting further investigation. To detect the chemical composition, gas chromatography-mass spectrometry was performed using an Agilent 6890N gas chromatograph, coupled with an Agilent 5973N mass selective detector and an HP-5MS capillary column. The injector and detector were preheated to temperatures of 240°C and 300°C, respectively. The oven temperature was then raised in programmed steps to 60°C, followed by 160°C, 450°C, and finally 600°C. The identification of compounds was done using retention indices and a mass spectral match with reference libraries. The EO of *C*. *zeylanicum* was found to be rich in cinnamaldehyde (64.14%) and eugenol (8.9%), while *E*. *globulus* predominantly contained eucalyptol (82.85%) and 1R-α-pinene (13.78%). The antimicrobial potential of the oils was determined using the agar well diffusion and broth microdilution methods. *Cinnamomum zeylanicum* and *E*. *globulus* showed inhibitory zones against *Salmonella* in an agar well diffusion assay at 26 ± 7.6 mm and 16 ± 6.8 mm, respectively. The minimum inhibitory concentrations against multidrug-resistant isolates of *Salmonella* were 64.1 ± 32.1 µg/ml for *C*. *zeylanicum* and 68.9 ± 32.9 µg/ml for *E*. *globulus*.

This experiment was conducted on day-old broiler chicks (*n* = 135) reared for 35 days in an experimental room designated at the Institute of Microbiology, University of Veterinary and Animal Sciences, Lahore. Antibiotic-free *ad libitum* feed and water were made available during the experiment. Birds were fed with two types of diets; the starter diet was provided till 21 days, followed by the finisher diet till the 35th day. The chicks were randomly divided into nine treatment groups, each containing 15 birds, as listed in Table 1. A randomized complete block design was used in the study. For the evaluation of anti-*Salmonella* activity, selected oils (*E*. *globulus* and *C*. *zeylanicum*) were administered in the feed of broiler birds from the 1st day. Test groups were challenged with SE(day 7) and SG(day 14) isolates that were isolated and characterized for their *in vitro* activities. Positive control groups received a bacterial challenge with ordinary feed, while the negative control (NC) group received no treatment. The study design is presented in Table 1. Chickens of all groups were vaccinated against ND and IBD according to the routine vaccination program for broilers. The ND vaccine includes the administration of a live virus “Lasota” vaccine via the eye drop route, followed by a booster dose, as listed in Table 1.

**Table 1.** Designated treatment groups (experiment design).

Experimental plan	Grouping	Treatment
NC	NC	Group with no treatment
Positive control	SE	Group fed on antibiotic-free feed and challenged with SE (1.5×108 CFU) at day 7.
	SG	Group fed on antibiotic-free feed and challenged with SG (1.5×108 CFU) at day 14
Preventive groups	SE+AB	Group fed on feed containing antibiotics and challenged with SE (1.5×108 CFU) at day 7.
	SG+AB	Group fed on feed containing antibiotics and challenged with SG (1.5×108 CFU) at day 14.
Treatment groups	SE+EG	Group challenged with SE and treated with E. globulus oil (1.5×108 CFU/ml), day 7.
	SE+CZ	Group challenged with SE and treated with C. zeylanicumm oil (1.5×108 CFU/ml), day 7.
	SG+EG	Group challenged with SG and treated with E. globulus (1.5×108 CFU/ml) on day 14,
	SG+CZ	Group challenged with SG and treated with C. zeylanicumm oil (1.5×108 CFU/ml), day 14.

NC: Group with no treatment; SE: Salmonella Enteritidis; SE+AB: Salmonella Enteritidis + Antibiotic; SE+EG: Salmonella Enteritidis + Eucalyptus globulus; SE+CZ: Salmonella Enteritidis + Cinnamomum zeylanicum; SG: Salmonella Gallinarum; SG+AB: Salmonella Gallinarum + Antibiotic; SG+EG: Salmonella Gallinarum + Eucalyptus globulus; SG+CZ: Salmonella Gallinarum + C. zeylanicum.

### Body weight (BW) gain

BW gain was measured weekly by weighing all birds in each treatment group. For monitoring feed intake (FI) and water consumption, data were collected daily [22]. The calculation of FI was performed by measuring the difference between the feed offered to the birds and the leftover feed [23].

### Feed conversion ratio (FCR)

The calculation of the FCR was done by dividing the feed consumed by the BW gained [23]. At the end of the week, FCR was calculated for each experimental group.

### Challenge with Salmonella strains

The strain used in this experiment was isolated in our previous study, and its *in vitro* activity against the EO was also investigated in our previous study [21,24]. The stored cultures were revived on *Salmonella*-Shigella agar. Birds were administered an oral dose of 10^8^ CFU of SEandSGon day 6. Cloacal swabs (*n* = 4) from each group were used to enumerate *Salmonella* before challenge and on day 15. While four birds from each group were slaughtered on day 35, the cecal contents were processed for enumeration. *Salmonella* counts were performed on freshly passed fecal samples using the serial dilution method and expressed as log10 colony-forming units per gram of dropping contents [25,26].

### Immunomodulatory effects against Newcastle disease virus (NDV)

The immunomodulatory effect of EOs in broiler chicks against NDV was determined every week (at 14, 21, 28, and 35 days) throughout the experiment by collecting blood and processing serum samples for the determination of serum antibody titers against NDV by using the hemagglutination inhibition assay [27].

### Antibody titer against infectious bursal disease virus

Antibody titers against IBD were determined on days 7, 14, 21, and 28 using an ELISA Synbiotic kit (San Diego, USA) according to the manufacturer’s guidelines.

### Analysis of intestinal morphometric parameters

After completion of the experimental duration, i.e., the 35th day, five (05)birds from each group were randomly selected, slaughtered for the collection of tissue samples (5 cm) from the small intestine (ileum, jejunum, and duodenum), and preserved in 10% formalin. Tissue sections (2 cm) of the small intestine were processed using paraffin embedding techniques, followed by staining of the prepared slides with hematoxylin and eosin. Different histomorphometric parameters of the small intestines, including villus width, villus height, crypt depth, and villus height ratio, as well as lamina propria width, were determined using Labomed Pixel Pro software. Images were captured at 4x magnification (Labomed microscope) with a microscopic camera. The measurements of each intestinal segment were calculated individually. The mean values were calculated with standard deviations (SD). The significance of the differences between the data was estimated using a *t*-test in SPSS version 20.

### Statistical analysis

Optical density measurements were presented as mean ± SD, and comparisons among experimental groups were made using one-way ANOVA followed by Tukey’s post hoc test. Similarly, *Salmonella* counts were reported as the mean ± SD in log_10_ CFU per gram or milliliter, and statistical analysis was performed using one-way ANOVA with Tukey’s test at a significance level of *p* < 0.05, employing SPSS software version 20.

## Results

### BW and FCR

**Figure 1.**
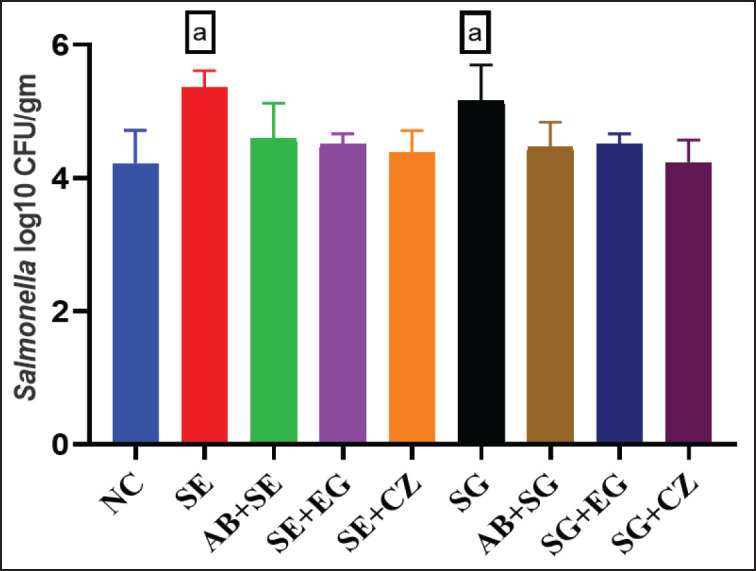
Effect of EOs on cloacal Salmonella count in broiler chicks challenged with SE and SG as determined by mean ± SD log10 CFU/gm on day 35. NC: Group with no treatment; SE: Salmonella Enteritidis; SE+AB: Salmonella Enteritidis + Antibiotic; SE+EG: Salmonella Enteritidis + Eucalyptus globulus; SE+CZ: Salmonella Enteritidis + Cinnamomum zeylanicum; SG: Salmonella Gallinarum; SG+AB: Salmonella Gallinarum + Antibiotic; SG+EG: Salmonella Gallinarum + Eucalyptus globulus; SG+CZ: Salmonella Gallinarum + Cinnamomum zeylanicum; a: statistically significant difference between control and challenge alone groups. *, **, *** Statistically significant difference between respective challenge group and treatment groups at p values ≤ 0.05, 0.005, and 0.001, respectively.

The impact of EO supplementation on the average BW of birds was assessed weekly, as illustrated in Table 2 and Figure 1. The results showed no statistically significant difference (*p* < 0.05) in mean BW between the control and treatment groups on days 1 and 7. Significantly higher BW (1,379.6 ± 83.59) was observed in the group challenged with SEand treated with *C. zeylanicum* (SE+CZ) oil compared with the control groups. The results of day 35 show that the group in which birds were challenged with SEand treated with *C*. *zeylanicum* oil has a significantly higher BW (1,650.9 ± 43.8 gm) compared to the groups treated with antibiotics and *E*. *globulus* oil. Furthermore, it was revealed that treatment groups, including antibiotic treatment (SE+AB), *E*. *globulus* treatment (SE+EG), and *C*. *zeyalinicum* treatment (SE+CZ), had significantly higher weights (1,587 ± 27.9, 1,562 ± 71.9, and 1,650.9 ± 43.8 gm, respectively) compared to the SEgroup (1,431.2 ± 74.4 gm). Similarly, groups treated with antibiotics (SG+AB), *E*. *globulus* treatment (SG+EG), and *C*. *zeyalinicum* treatment (SG+CZ) had significantly higher weights (1,504.8 ± 70.5, 1,586.5 ± 49.65, and 1,627 ± 51.2 gm, respectively) compared to the group challenged with SG(1,360.5 ± 58.4 gm). The effect of *C*. *zeylanicumm* on the weight gain of broilers was significantly higher as compared to the effect of *E*. *globulus* in broilers challenged with SE(1,650.9 ± 43.8 *vs.* 1,562 ± 71.9 gm) and non-significantly higher in broilers challenged with SG(1,627 ± 51.2 *vs.* 1,586.5 ± 49.65 gm). The effect of EO on the FCR is presented in Table 2, which indicates that the NC group had a better FCR (1.87) compared to the SE and SG challenge (1.92 and 1.97, respectively). Groups treated with antibiotics, *E*.* globulus,* and *C*.* zeylanicum* after challenge with SEand SG had better FCR as compared to the respective challenge group. A higher FCR was observed in groups treated with *C*.* zeylanicum* oil and *E*.* globulus* oil.

**Table 2.** Effect of *E. globulus* and *C. zeylanicum* on BW, FCR, and *Salmonella* count.

Parameter	Day	NC	SE	SE+AB	SE+EG	SE+CZ	SG	SG+AB	SG+EG	SG+CZ
Mean BW (gm)	0 day	40.7 ± 0.67a	41 ± 0.66a	40.9 ± 0.73a	41.3 ± 0.48a	41.3 ± 0.48a	41 ± 0.66a	40.6 ± 0.51a	41 ± 0.66a	41.1 ± 0.56a
	7th day	144.4 ± 9.32a	140.2 ± 6.54a	154.2 ± 7.05a	145.8 ± 8.77a	140.1 ± 11.1a	139.7 ± 1.49a	150.7 ± 10.49a	144.4 ± 3.89a	151.5 ± 7.48a
	14th day	412 ± 17.5a	335.7 ± 57.0b	415 ± 26.3a	426.9 ± 26.67a	447.7 ± 31.1a	367.5 ± 61.84b	399 ± 20.24a	398 ± 48.02a	431.8 ± 20.8a
	21st day	743.9 ± 50.4a	783.1 ± 35.9a	732.4 ± 62.a	793 ± 54.07a	821 ± 22.25b	701 ± 67.45a	787.2 ± 74.4a	838.7 ± 35.7b	817.2 ± 16.6b
	28th day	1,213.8 ± 61.23a	1,168 ± 61.48a	1,236 ± 42.7a	1,264.8 ± 71.9a	1,379.6 ± 83.59b	1,172.8 ± 29.1a	1,267.9 ± 35.9c	1,257.8 ± 26.79c	1,356 ± 95.0b
	35th day	1,432.9 ± 49.3a	1,431.2 ± 74.4a	1,587 ± 27.9b	1,562 ± 71.9b	1,650.9 ± 43.8c	1,360.5 ± 58.4a	1,504.8 ± 70.5a	1,586.5 ± 49.65c	1,627 ± 51.2c
FCR	day 7	1.01	1.05	1	1	1.03	1.01	1	1.01	1
	day 14	1.2	1.22	1.2	1.18	1.13	1.2	1.21	1.2	1.15
	day 21	1.46	1.49	1.44	1.44	1.4	1.55	1.48	1.44	1.4
	day 28	1.54	1.57	1.53	1.5	1.5	1.6	1.49	1.49	1.45
	day 35	1.87	1.93	1.83	1.74	1.73	1.97	1.8	1.76	1.75
Salmonella count (Mean log10CFU/gm ± S.D)	7th day	2.53 ± 0.07	2.58 ± 0.12	2.52 ± 0.11	2.55 ± 0.1	2.52 ± 0.55	2.65 ± 0.12	2.52 ± 0.11	2.58 ± 0.12	2.52 ± 0.37
	8th day	3.17 ± 0.23	3.21 ± 0.22	3.11 ± 0.12	3.07 ± 0.1	2.96 ± 0.07	3.07 ± 0.14	3.04 ± 0.06	2.92 ± 0.11	2.93 ± 0.36
	10th day	3.53 ± 0.31	4.31 ± 0.24	4.19 ± 0.27	4.07 ± 0.24	3.95 ± 0	3.4 ± 0.36	3.52 ± 0.13	3.39 ± 0.11	3.3 ± 0.37
	14th day	4.7 + 1.95	4.8 + 0.91	4.62 + 0.87	4.36 + 1.19	4.25 + 0.73	4.53 + 0.99	4.52 + 0.97	3.99 + 1.05	3.95 ± 0
	17th day	4.01 + 1.08	5.05 + 0.41	4.96 + 0.67	4.53 + 0.97	4.41 + 0.55	4.83 + 0.46	4.86 + 0.50	4.86 + 0.50	4.86 ± 0.50
	21st day	4.3 ± 1.05	5.46 ± .23	5.23 ± 0.57	4.9 ± 0.55	4.72 ± 0.76	5.16 ± 0.54	5.08 ± 0.44	5.03 ± 0.4	4.86 ± 0.5
	28th day	4.4 ± 0.58	5.31 ± 0.44	5.03 ± 0.57	4.77 ± 0.5	4.74 ± 0.43	5.1 ± 0.47	4.93 ± 0.41	4.78 ± 0.33	4.6 ± 0.69
	35th day	4.22 ± 0.491a	5.35 ± 0.255b	4.58 ± 0.53a	4.5 ± 0.15a	4.38 ± 0.32a	5.157 ± 0.54b	4.46 ± 0.37a	4.5 ± 0.15a	4.23 ± 0.33

NC: Group with no treatment; SE: Salmonella Enteritidis; SE+AB: Salmonella Enteritidis + Antibiotic; SE+EG: Salmonella Enteritidis + Eucalyptus globulus; SE+CZ: Salmonella Enteritidis + Cinnamomum zeylanicum; SG: Salmonella Gallinarum; SG+AB: Salmonella Gallinarum + Antibiotic; SG+EG: Salmonella Gallinarum + Eucalyptus globulus; SG+CZ: Salmonella Gallinarum + Cinnamomum zeylanicum; ns: statistically non-significant difference between control and challenge-alone groups. a, b, c Values in different rows of the same column with a different superscript differ significantly.

### Effect of EOs on Salmonella count

The findings showed that broiler birds infected with SEhad a significantly elevated bacterial load compared to the NC group (5.35 ± 0.255 log_10_*vs.* 4.22 ± 0.491 log_10_ CFU/gm). *Salmonella* count was significantly decreased (*p* < 0.05) in groups challenged by SEthen treated with *C*.* zeylanicum* (4.38 ± 0.32 log_10_ CFU/gm) oil as compared to groups challenged with SEalone, and non-significantly lower as compared to groups treated with *E. globulus* (4.5 ± 0.15 log_10_ CFU/gm) and antibiotics (4.58 ± 0.53 log_10_ CFU/gm). There was a non-significant difference in *Salmonella* counts between groups treated with *C*.* zeylanicum* (4.38 ± 0.32 log_10_ CFU/gm) oil and *E*.* globulus* (4.5 ± 0.15 log_10_ CFU/gm). Similarly, broiler chickens exposed to SG exhibited a higher bacterial count than those in the NC group (5.157 ± 0.54 log_10_ *vs.* 4.22 ± 0.491 log_10_ CFU/gm). Results indicated that this increase in *Salmonella* count was significantly ameliorated in groups treated with *C*.* zeylanicum* oil (4.23 ± 0.33 log_10_ CFU/gm) and non-significantly in *E*.* globulus* oil (4.5 ± 0.15 log_10_ CFU/gm) and antibiotics (4.46 ± 0.37 log_10_ CFU/gm), as presented in Table 2.

### Antibody titer against ND and IBD

The geometric mean titers (GMTs) of birds against the NDV vaccine were significantly higher in groups given different treatments on days 14, 21, 28, and 35 when compared with control groups, as presented in Table 3. The highest titer against live vaccine of ND was obtained on day 28, at 96 and 95.8 in the treatment groups (SE+CZ) and (SG+CZ), respectively. The lowest NDV titer was observed on day 35 in the NC group and the groups challenged with SE and SG. At Day 35, GMT against the NDV vaccine in different groups of birds (NC, SE, SE+AB, SE+EG, SE+CZ, SG, SG+AB, SG+EG, and SG+CZ) was 24.15 and 31.9. 44.6. 63.7, 82.6, 36, 44.6, 72.5, and 84, respectively. Results revealed that birds given *E*.* globulus* and *C*.* zeyalinicum* had a better GMT against the NDV vaccine compared to other groups. The mean antibody titer of different treatment groups of chicks in various experimental groups against the IBD Virus vaccine at days 21, 28, and 35 is presented in Table 3. At Day 35, the mean antibody titer against the IBD vaccine in different groups of birds (NC, SE, SE+AB, SE+EG, SE+CZ, SG, SG+AB, SG+EG, and SG+CZ) was 3,260 ± 71.6, 3,281.25 ± 56.6, 3,520 ± 48.9, 3,367.5 ± 95.3, 4,074.5 ± 969.2, 3,192.5 ± 57.3, 3,390 ± 87.5, 4,299 ± 1,546.8, and 4,947 ± 2,447, respectively. Antibody titer was non-significantly higher in groups given *E*.* globulus* (SE+EG; SG+EG) and *C*.* zeylanicumm* (SE+EG; SG+EG) as compared to the NC group. At day 35, group SG+CZ had a significantly higher (*p* ≤ 0.05) titer (4,947 ± 2,447) against the IBD vaccine compared to the respective challenge group (3,260 ± 71.6), as shown in Table 3.

**Table 3.** Immunomodulatory effect of *E. globulus* and *C. zeylanicum* on GMT against the NDV vaccine and antibody titers against IBD virus of chicks on different days.

Parameter	Day	NC	SE	SE +AB	SE+EG	SE+CZ	SG	SG +AB	SG+EG	SG+CZ
GMT against the NDV vaccine	14th day	13.1	13.1	22.38	15.8	38	15.8	15.8	26.3	31.62
	21st day	31.6	31.6	44.66	38	44.6	26.6	44.66	31.6	44.6
	28thday	42.2	36.74	47.8	86	96	42.2	48.48	88	95.8
	35thday	24.15	31.9	44.6	63.7	82.6	36	44.6	72.5	84
Antibody titer against IBD vaccine (Mean ± SD)	21st day	3,195 ± 34.1a	3,242.5 ± 99.4a	3,432.5 ± 49.2a	3,247.5 ± 103a	3,194.5 ± 492.4a	3,135 ± 133.7a	3,347.5 ± 97a	3,382.5 ± 75a	3,478.25 ± 280.4a
	28thday	3,260 ± 158.1a	3,215 ± 85.4a	3,375 ± 45a	3,374.5 ± 81a	3,395 ± 151.1a	3,070 ± 183.4a	3,262.5 ± 120.3a	3,377.5 ± 113.5a	3,607.5 ± 374.5a
	35thday	3,260 ± 71.6a	3,281.25 ± 56.6a	3,520 ± 48.9a	3,367.5 ± 95.3a	4,074.5 ± 969.2a	3,192.5 ± 57.3a,b	3,390 ± 87.5a	4,299 ± 1546.8a	4,947 ± 2447a,c

NC: Group with no treatment; SE: Salmonella Enteritidis; SE+AB: Salmonella Enteritidis + Antibiotic; SE+EG: Salmonella Enteritidis + Eucalyptus globulus; SE+CZ: Salmonella Enteritidis + Cinnamomum zeylanicum; SG: Salmonella Gallinarum; SG+AB: Salmonella Gallinarum + Antibiotic; SG+EG: Salmonella Gallinarum + Eucalyptus globulus; SG+CZ: Salmonella Gallinarum + Cinnamomum zeylanicum. a, b, c Values in different rows of the same column with a different superscript differ significantly.

### Histomorphometric parameters of the small intestine

**Figure 2.**
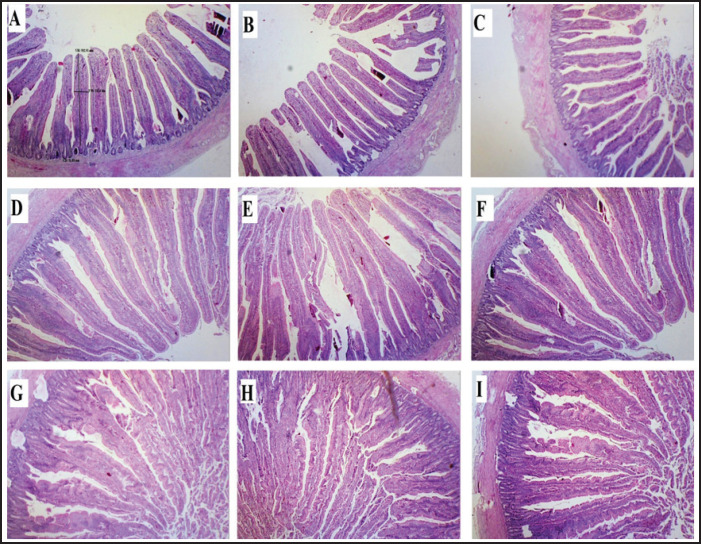
Effects of C. zeylanicum on gut morphology in broiler chicks challenged with SE. Cross-section photograph of ileum A = Antibiotic + Salmonella Enteritidis, B = control broilers, C = Salmonella Enteritidis + Cinnamomum zeylanicum; cross-section photograph of Jejunum, D = control broilers, E = Antibiotic + Salmonella Enteritidis, F = Salmonella Enteritidis + Cinnamomum zeylanicum; cross-section photograph of Duodenum, G = Control broilers, H = Antibiotic + Salmonella Enteritidis, I = Salmonella Enteritidis + Cinnamomum zeylanicum.

Histomorphometric measurements of the ileum, jejunum, and duodenum are presented in Table 4, and the histological morphology of different parts of the gut is presented in Figure 2. The birds’ supplementation with EOs of *C*.* zeylanicum* and *E*.* globulus* increased villus height and villus surface area in the ileum, jejunum, and duodenum sections of the small intestines when compared to the NC.

**Table 4.** Effect of *e. globulus* and *c. zeylanicum* on intestine morphology in broiler chicks.

	NC	SE	SE+AB	SE+EG	SE+CZ	SG	SG+AB	SG+EG	SG+CZ
Duodenum									
Villus height (µm)	1,031 ± 9.30a	1,032.25 ± 14.88a	839 ± 4.11b	1,237 ± 5.71c	1,125.5 ± 13.20d	825.5 ± 45.23b	801.5 ± 4.43b	1,212.5 ± 24.51c	1,161.75 ± 2.5d
Villus width (µm)	150.5 ± 9.98a	141 ± 5.77a	139.5 ± 4.99a	192.25 ± 3.30b	179.5 ± 6.60b	116.5 ± 7.88c	116 ± 4.43c	183.5 ± 4.72b	176.5 ± 3.8b
Villussurface area(mm2)	0.48 ± 0.03a	0.45 ± 0.01a	0.48 ± 0.01a	0.74 ± 0.01b	0.63 ± 0.01b	0.43 ± 0.01a	0.41 ± 0.01a	0.69 ± 0.02b	0.63 ± 0.0b
Crypt depth (µm)	165.5 ± 4.43a	177.2 ± 13.4a	95.75 ± 3.30b	144.5 ± 4.12c	130.5 ± 7.5d	82.5 ± 6.21e	80 ± 6.73e	139.5 ± 7.72c	133.75 ± 11.08c
Villus height: crypt depth	6.22 ± 0.12a	5.84 ± 0.42a,b	5.48 ± 0.07 a,b	8.56 ± 0.25c	8.64 ± 0.46c	6.21 ± 0.37a	5.89 ± 0.23a	8.71 ± 0.62c	8.72 ± 0.75c
Jejunum									
Villus height (µm)	1,007 ± 13.71a	1,116.25 ± 3.30b	1,131.25 ± 14.22b	831.5 ± 35.34c	801.5 ± 10.87c	839 ± 8.40c	974.5 ± 16.05d	825.5 ± 12.28c	817 ± 5.29c
Villus depth(µm)	125 ± 11.37a	135.5 ± 7.72a,b	144.75 ± 6.70b,c	117 ± 9.30a	116 ± 11.43a	139.5 ± 4.50 a,b	144.5 ± 3.87 b,c	116.5 ± 6.45a	111 ± 11.8a
Villus surface area(mm2)	0.39 ± 0.03a	0.47 ± 0.02 a	0.51 ± 0.02 a	0.2975 ± 0.03a	0.29 ± 0.02 a	0.36 ± 0.01 a	0.28 ± 0.02a	0.3 ± 0.018 a	0.28 ± 0.02 a
Crypt depth(µm)	106.5 ± 7.72a	120.5 ± 3.4b	125.25 ± 2.5b	86.75 ± 3.40c	80 ± 7.83c	95.75 ± 1.70d	111.75 ± 6.13a	82.5 ± 4.43c	77.5 ± 8.22c
Villus height: crypt depth	9.48 ± 0.6a	9.26 ± 0.28a	9.03 ± 0.21a	9.6 ± 0.8a	10 ± 1.15a	8.76 ± 0.12b	8.74 ± 0.62b	10 ± 0.6a	10.63 ± 1.18c
Ileum									
Villus height (µm)	610.5 ± 34.91a	574.25 ± 63.1a,b	533 ± 9.41b	792.75 ± 1.70c	862.75 ± 1.70d	604.5 ± 12.87a	521.5 ± 15.96b	779.5 ± 9.67c	851 ± 6.21c,d
Villus width (µm)	83.5 ± 9.03a	102 ± 7.52b	80.75 ± 5.25a	113.5 ± 4.08b,c	118.5 ± 3.4c	86.5 ± 3.87a	77 ± 4.08a	97 ± 1.91a,b	96.25 ± 8.65a,b
Villus surface area (mm2)	0.155 ± 0.019a,b	0.187 ± 0.022a,b	0.132 ± 0.01a	0.275 ± 0.005c	0.312 ± 0.009	0.16 ± 0.008a,b	0.122 ± 0.005a	0.23 ± 0.014d	0.252 ± 0.026c,d
Crypt depth (µm)	81.5 ± 3.87a,b	70.5 ± 16.82a,b	73.5 ± 4.79a,b	98.5 ± 5.44c,d	102 ± 2.16c,d	83.5 ± 4.43a.b,c	79 ± 5.47a,b	97 ± 2.44a,c,d	98 ± 2.94c,d
Villus height: crypt depth	6.62 ± 0.52a	8.42 ± 1.67b	7.93 ± 0.25a	7.25 ± 0.46a,c	8.455 ± 0.193b	7.48 ± 0.38c	7.27 ± 0.5a,c	8.06 ± 0.45b,c	8.685 ± 0.228b

NC: group with no treatment; SE: salmonella Enteritidis; SE+AB: salmonella Enteritidis + antibiotic; SE+EG: salmonella Enteritidis + eucalyptus globulus; SE+CZ: salmonella Enteritidis + cinnamomum zeylanicum; SG: salmonella Gallinarum; SG+AB: salmonella Gallinarum + antibiotic; SG+EG: salmonella Gallinarum + eucalyptus globulus; SG+CZ: salmonella + cinnamomum zeylanicum; NS: statistically non-significant difference between control and challenge alone groups. a, b, c, d, e values in different rows of the same column with a different superscript differ significantly.

The histomorohic measurements of the ilium are indicated in Table 4. Among the* Salmonella* Enteritidis-challenged birds’ group (SE+CZ), the maximum increase in villus surface area (0.312 ± 0.009) and villus height was observed, followed by the group (SE+EG). These are the* C*.* zeylanicum* and *E*.* globulus* oil-treated groups, respectively. Similarly, among SG-challenged birds, the group supplemented with *C*.* zeylanicum* oil exhibited a significantly greater surface area (0.252 ± 0.026) compared to the *E*.* globulus* and antibiotic-treated groups (0.122 ± 0.005 and 0.238 ± 0.014, respectively). The villus height-to-crypt depth ratio was significantly elevated in the (SG+CZ) treatment group.

Similarly, supplementation with *C*. *zeylanicum* and *E*.* globulus* oil increased histomorphometric measurements of the jejunum, revealing that the antibiotic-treated group showed the maximum villus surface area (144.75 ± 6.70) compared to other treatment groups.The* C*. *zeylanicum*-treated group (SG+CZ) has the highest villus height-to-crypt depth ratio, as shown in Table 4.

While comparing the effect of *C*.* zeylanicum* and *E*.* globulus* on morphometric measurements of the duodenum, (SE+EG) had maximum villus surface area (0.74 ± 0.01) and villus height (1,131.25 ± 14.22) as compared to all other experimental groups. The group supplemented with* C*.* zeylanicum* oil (SG+CZ) exhibited a statistically significant difference compared to the control group. Histometromorphic measurements of the duodenum are shown in Table 4. The groups supplemented with *C*.* zeylanicum and E*.* globulus* had a statistically significant effect on duodenum villus height and crypt depth ratio.

## Discussion

In the current study, challenging birds with SEhas affected the birds’ performance. *Salmonella* primarily resides in the caecum of poultry birds and then spreads to other major organs through both circulatory and lymphatic routes [28]. In addition, *Salmonella* colonizes the reproductive organs of layers, from which it contaminates the eggs [29]. Our results from the poultry bird trial indicate that administering EO in feed significantly reduces *Salmonella* colonization. In various studies, the oral route has been used to challenge broiler birds with *Salmonella*. It is suggested as a valuable model for assessing the efficacy of different therapeutics, yielding similar results in terms of reducing *Salmonella* colonization [30,31]. There is a contradiction in the literature regarding the effect of *Salmonella* administration on growth performance. According to Vandeplas et al. [32], exposing broiler chickens to *Salmonella* led to a notable decline in their growth performance. In contrast, another study revealed that challenging broiler birds with *Salmonella* did not affect their performance [33]. The deteriorated growth performance may be attributed to the fact that the birds’ FI has been reduced due to damage to their intestinal musculature. Variations in findings across different studies may be attributed to the specific *Salmonella* strains or inoculation doses used, as well as the bird species involved, which can influence the extent of intestinal mucosal damage. Relying solely on feed additives is not sufficient to completely eradicate *Salmonella* infections. Nonetheless, a reduction in *Salmonella* load can significantly enhance the microbiological safety of poultry feed.

The application of *C*.* zeylanicum* EO presents a promising strategy to limit *Salmonella* colonization. Controlling *Salmonella* in poultry is particularly important for reducing the incidence of foodborne illness in humans linked to poultry meat consumption. Compounds found in plant-derived oils, such as trans-cinnamaldehyde and eugenol, have demonstrated strong antimicrobial effects against *Salmonella* in both broiler and layer chickens [25]. The results of our study proved that broilers supplemented with *C*.* zeylanicum* oil had a reduced number of *Salmonella* colonies. In the present study, supplementation with *C*.* zeylanicum* resulted in the highest average daily weight gain and improved FCR, aligning with the findings reported by Ciftci et al. [34]. These outcomes are in agreement with previous studies that documented enhanced growth performance and reduced intestinal *Salmonella* levels in broilers receiving *C*.* zeylanicum* supplementation [35,36]. Additionally, various *in vitro* investigations have demonstrated the antibacterial activity of EOs against *Salmonella*. Notably, oils derived from bay, thyme, clove, and Cinnamon exhibited strong inhibitory effects on several major foodborne pathogens, including *Escherichia coli*, SE, *Campylobacter jejuni*, *Staphylococcus aureus*, and *Listeria monocytogenes* [37,38].

The replacement of antibiotic growth promoters with natural plant extracts can be a valuable tool for both the poultry industry and humans. To maintain and improve the general basic health of poultry birds, EOs can play a supportive role as single or mixed preparations. EOs can improve the physiology of the digestive system, enhance blood circulation, exhibit antioxidant properties, reduce the intensity of pathogenic microbes, and effectively invigorate the immune status of poultry birds. In contrast to our findings, some other studies reported that cinnamon oil supplementation in feed did not show any impact on BW gain, FCR, and F) of broiler birds. Various doses of *C*.* zeylanicum* powder (250, 500, 1,000, or 2,000 mg/kg) or its EO showed no significant impact on the feed consumption or growth performance of broiler chickens [39]. Barreto et al*.* [40]have reported similar findings after the integration of *C*.* zeylanicum* extract (1,000 ppm) into the diet of poultry birds. The average growth performance of the broiler birds was also not affected after the supplementary diet with the cinnamaldehyde.

Moreover, a blend of EOs of clove and Cinnamon in the diet of broiler birds makes no difference in birds’ performance [41]. Nonetheless, adding a 200 ppm blend of EOs from oregano, Cinnamon, and pepper to the diet led to an improvement in FCR [42]. The reasons behind the inconsistencies observed in various study outcomes remain uncertain; however, they may stem from variations in the dosage of *C*.* zeylanicum* oil administered, as well as differences in the concentration of key bioactive constituents, such as cinnamaldehyde and eugenol, present in different parts of the plant, like bark, leaves, or flowers. Additional contributing factors could include the duration of oil supplementation, the specific broiler breed used, and the overall feeding regime.

Furthermore, discrepancies in the effectiveness of *C*.* zeylanicum* oil may also be influenced by factors such as the nutritional composition of the basal diet, the birds’ daily FI, environmental parameters, and the level of farm hygiene. For instance, diets formulated with highly digestible ingredients can reduce bacterial growth in the gut, potentially limiting the antimicrobial efficacy of phytogenic additives. Barbour et al. [43] reported that *E*.* globulus* EO enhanced weight gain in broilers affected by respiratory infections. Another study reported that the EO of* E*.* globulus* shows antimicrobial and immunostimulatory effects in broilers [44]. Our results also do not align with those of a few previous studies regarding the impact of *Eucalyptus* oil on weight gain and FCR during the 1–42 day period [45,46].

EOs of various medicinal plants can enhance poultry production, improve immune response, and lead to higher antibody production against different diseases in poultry [47,48]. Our results indicated that *Eucalyptus* and Cinnamoncan both modulate the immune response against the NDV vaccine in broilers challenged with SEor SG. EO of Cinnamon was a better immune enhancer compared to *Eucalyptus.* The antimicrobial properties of Cinnamon and *Eucalyptus* have been previously reported as well [47-50].

The birds’ supplementation with EOs of *C*.* zeylanicum* and *E*.* globulus* increased villus height and villus surface area in the ileum, jejunum, and duodenum sections of the small intestines when compared to the NC. Similar results have been reported in a study that found EOs from *C*.* zeylanicum* and *E*.* globulus* exhibited remarkable effectiveness against *Salmonella*, contributing to improved small intestine morphology and enhancing nutrient absorption [51]. Among theSE-challenged birds’ group (SE+CZ), there was a maximum increase in villus surface area and villus height, followed by the group (SE+EG).

These are the* C. zeylanicum* and *E*.* globulus* oil-treated groups, respectively. Similarly, among SG-challenged birds, the group supplemented with *C*.* zeylanicum* oil exhibited a significantly greater surface area compared to the *E*.* globulus* and antibiotic-treated groups, respectively. The Villus height crypt depth ratio was considerably higher in the (SG+CZ) group. A similar study was conducted to investigate the effect of EO delivery routes on the intestinal morphology of broilers, revealing that the overall mucosal thickness of the ileum was enhanced in the treated groups [52]. The addition of *C*.* zeylanicum* and *E*.* globulus* oils to the diet resulted in improved jejunal histomorphology; however, the group fed antibiotics had the highest villus surface area among all treatment groups. The ratio of villus height and crypt depth is the highest in the *C*.* zeylanicum*-treated group. A research study conducted to investigate the impact of a blend of various EOs on the intestines of broilers revealed that the oils enhanced intestinal development, protected microvilli, and stimulated the release of endogenous enzymes [53]. *Cinnamomum zeylanicum* and *E*.* globulus* supplemented groups were found to have significant positive effects on duodenum villus height and crypt depth ratio. Another study found a positive and significant effect of the EO combination on the height of the duodenal villi in broilers. It was demonstrated that the treatment enhanced growth performance in birds, attributed to increased digestion and intestinal absorption, as observed by morphology [54,55].

Although this current study provides promising results on the antibacterial effects of *C*. *zeylanicum* and *E*. *globulus* EOs in broilers, one of the drawbacks of the study is that a single replicate pen was used per treatment due to resource constraints. Notwithstanding this, the trial has provided useful preliminary information regarding the potential of *C*. *zeylanicum* and *E*. *globulus* EOs against *Salmonella* in broilers. To augment these findings, additional research should be conducted with a larger number of replicates and similar sample sizes to further reinforce the results.

## Conclusion

In conclusion, *C. zeylanicum* EO and *E. globulus* EO were found to have significant *in vivo* anti-*Salmonella* activity. Based on these findings, it is recommended that such EOs be further explored and potentially developed as commercial alternatives to antibiotics for managing *Salmonella* in poultry production.

## List of abbreviations

AB, antibiotic; CZ, *Cinnamomum zeylanicum*; CFU, colony-forming unit; ELISA, enzyme-linked immunosorbent assay; EO, essential oil; EG, *Eucalyptus globulus*; FCR, feed conversion ratio; GMT, geometric mean titer; IBD, infectious bursal disease; IBDV, infectious bursal disease virus; NC, negative control; ND, Newcastle disease; NDV, Newcastle disease virus; SE, *Salmonella Enteritidis*; SG, *Salmonella Gallinarum*; SPSS, Statistical Package for the Social Sciences.
